# Unusual extension of a dental abscess through the pterygomandibular space in subcutaneous maxillofacial tissue

**DOI:** 10.1002/ccr3.5316

**Published:** 2022-01-24

**Authors:** Soultana Foutzitzi, Konstantinos Frigkas, Despoina Vitsa, Savas P. Deftereos

**Affiliations:** ^1^ Radiology Department D.U.Th University Hospital of Alexandroupolis Alexandroupolis Greece; ^2^ ENT Head and Neck Surgery Department D.U.Th University Hospital of Alexandroupolis Alexandroupolis Greece

**Keywords:** buccal space, computed tomography, infection, mandible, ultrasound

## Abstract

Infections of maxillary teeth commonly spread to the buccal space, whereas infections originating in the mandible usually spread to the submandibular, pterygomandibular, and buccal spaces. The pterygomandibular space may serve as a path for spread to zygomatic areas and thus must always be a part of imaging evaluations.

## Graphical abstract

X‐ray examinations and ultrasound studies of patients with maxillofacial emergency are common, and could be useful particularly in cases with severe and deep inflammatory processes. Beyond detecting the presence of air, CT scanning is useful for the evaluation of pathological extension. Thus, physicians must be familiar with the maxillofacial space.

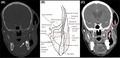

This article reports the case of a 68‐year‐old man admitted to the emergency department with a prominent swelling of the left cheek and submandibular and temporal extension, which had started 8 days earlier and gradually worsened (Figure 1A). In addition, mild trismus (restriction of mouth opening) was observed. Poor oral hygiene was identified through oral cavity examination. According to the patient's recent and/or past medical history, no dental or other maxillofacial pathologies were identified. Furthermore, the patient was receiving treatment for type 2 diabetes mellitus. Laboratory tests were consistent with infection (WBC: 23.000 k/ml with 82.1% neutrophils and CRP: 39 mg/dl). The presence of prominent, extended subcutaneous emphysema in the left temporomandibular space was observed through skull X‐ray. In addition, a soft‐tissue swelling with liquid‐gas level in it was present (Figure 1B,C). Ultrasound scans Figure 2(A,B) revealed a fluidlike collection with air bubbles (multiple moveable internal echoes), which was located between the mandibular bone and the masseter muscle, and extended through the superficial temporal space. Τhe possibility of a deeper abscess was difficult to exclude. Computed tomography (Figure 3) revealed a large abscess between the masseter muscle and the maxilla, and surrounding the maxilla. In addition, an extension through the submasseteric and pterygomandibular spaces to the superficial temporal space was observed. An impacted wisdom tooth (revealed by CT) was considered to be the cause of inflammation.^1^ The patient was treated with fission and drainage of the abscess cavity (removing approximately 55 cc of pus), and *Streptococcus* *anginosus* was isolated in the pus culture. Antibiotic treatment was administered, and the patient was discharged in good condition after 3 days. The patient presented after 7 days for a follow‐up examination and was found to be in healthy condition.

**FIGURE 1:**
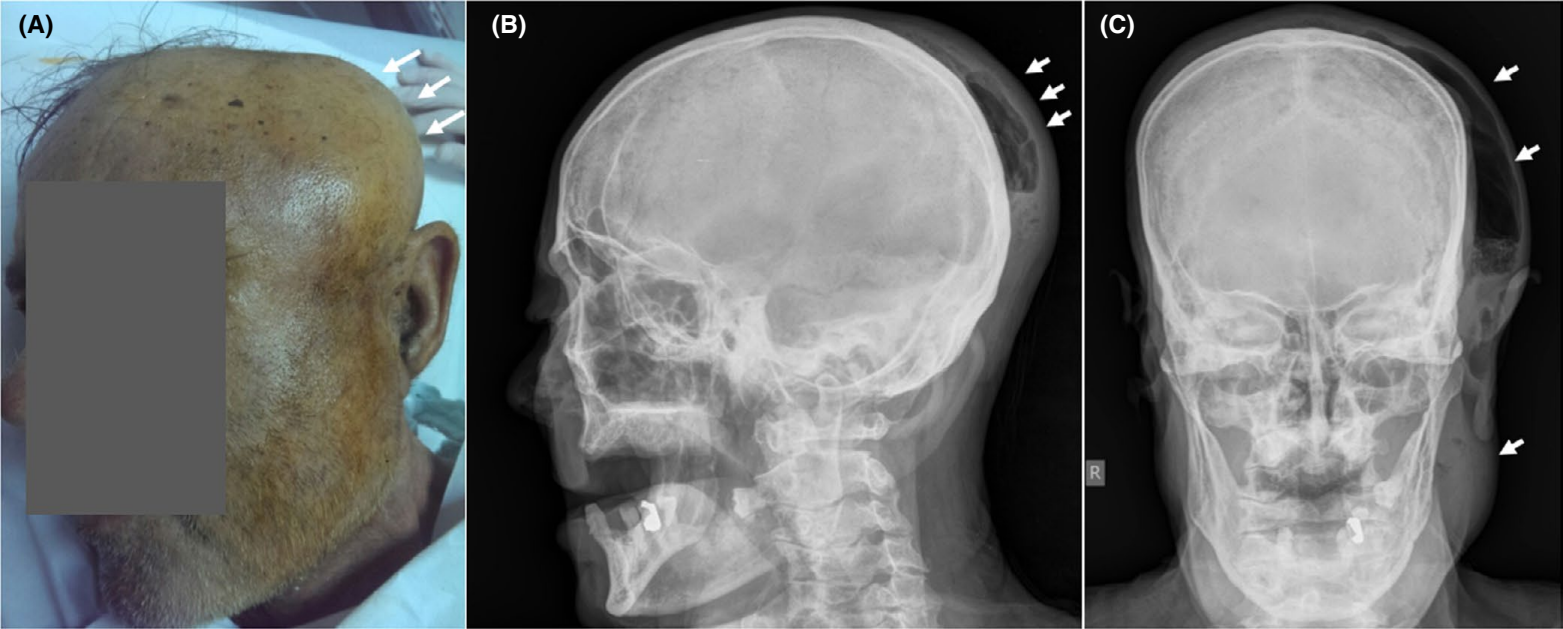
(A) A 68‐year‐old man with the presence of a prominent swelling of the left cheek with temporal and submandibular extension. (B, C) skull X‐rays (profile and posteroanterior projections, respectively). The presence of liquid‐gas level and swelling of soft tissues are obvious (arrows)

**FIGURE 2:**
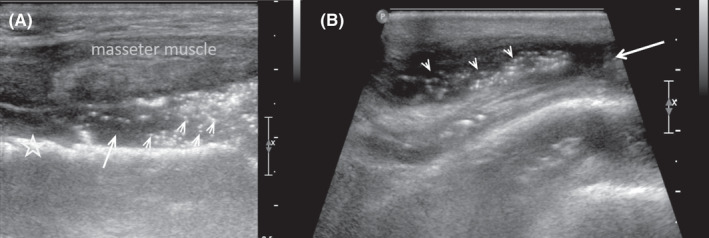
(A) Fluid collection (long arrows) with air bubbles as multiple internal reflections (small arrows) between the mandibular bone (star) and the masseter muscle, and (B) extension of the collection to the superficial temporal space

**FIGURE 3:**
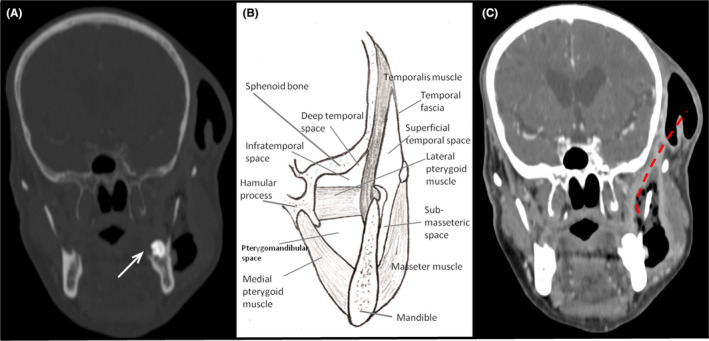
(A) CT, in coronal plane and «bone window». An impound wisdom tooth, which was considered the cause of inflammation, is noticed (arrow). (B) Drawing of the maxillofacial spaces. (C) The dissemination path (dashed line)

## CONFLICT OF INTEREST

None.

## AUTHOR CONTRIBUTIONS

Foutzitzi S: Had the conception and revised the work; she approved the final version and agreed to be accountable for all aspects of the work. Frigkas K: Made a draft and analyzed the data of the work; she approved the final version and agreed to be accountable for all aspects of the work. Vitsa D: Made a draft and interpreted the data of the work; he approved the final version and agreed to be accountable for all aspects of the work. Deftereos S: Designed and critically revised the work; he approved the final version and agreed to be accountable for all aspects of the work.

## Ethical Approval

I testify on behalf of all co‐authors that our article submitted to Clinical Case Reports.

## CONSENT

Written informed consent was obtained from the patient to publish this report in accordance with the journal's patient consent policy.

## Data Availability

The data that support the findings of this study are openly available in Radiology Department of DUTH ALEXANDROUPOLIS Greece, reference number 2005210014/1674594/21/05/2020.
